# Synthesis and NMR-Study of 1-Trimethylsilyl Substituted Silole Anion [Ph_4_C_4_Si(SiMe_3_)]^−^•[Li]^+^ and 3-Silolenide 2,5-carbodianions {[Ph_4_C_4_Si(*n*-Bu)_2_]^−2^•2[Li]^+^, [Ph_4_C_4_Si(*t*-Bu)_2_]^−2^•2[Li]^+^} via Silole Dianion [Ph_4_C_4_Si]^−2^•2[Li]^+^

**DOI:** 10.3390/molecules180910568

**Published:** 2013-08-30

**Authors:** Jang-Hwan Hong

**Affiliations:** Department of Nanopolymer Material Engineering, Pai Chai University, 155-40 Baejae-ro (Doma-Dong), Seo-Gu, Daejon 302-735, Korea; E-Mail: jhong@pcu.ac.kr; Tel.: +82-42-520-5755; Fax: +82-42-520-5798

**Keywords:** silacyclopentadiene, silole, anion, dianion, silyation, 3-silolenide, aromaticity, NMR

## Abstract

1-Trimethylsilyl, 1-R (R = Me, Et, *i*-Bu)-2,3,4,5-tetraphenyl-1-silacyclopentadiene [Ph_4_C_4_Si(SiMe_3_)R] are synthesized from the reaction of 1-trimethylsilyl,1-lithio-2,3,4,5-tetraphenyl-1-silacyclopentadienide anion [Ph_4_C_4_SiMe_3_]^−^•[Li]^+^ (**3**) with methyl iodide, ethyl iodide, and *i*-butyl bromide. The versatile intermediate **3** is prepared by hemisilylation of the silole dianion [Ph_4_C_4_Si]^−^^2^•2[Li]^+^ (**2**) with trimethylsilyl chloride and characterized by ^1^H-, ^13^C-, and ^29^Si-NMR spectroscopy. 1,1-bis(R)-2,3,4,5-tetraphenyl-1-silacyclopentadiene [Ph_4_C_4_SiR_2_] {R = *n*-Bu (**7**); *t*-Bu (**8**)} are synthesized from the reaction of **2** with *n*-butyl bromide and *t*-butyl bromide. Reduction of **7** and **8** with lithium under sonication gives the respective 3-silolenide 2,5-carbodianions {[Ph_4_C_4_Si(*n*-Bu)_2_]^−^^2^•2[Li]^+^ (**10**) and [Ph_4_C_4_Si(*t*-Bu)_2_]^−^^2^•2[Li]^+^ (**11**)}, which are characterized by ^1^H-, ^13^C-, and ^29^Si-NMR spectroscopy. Polarization of phenyl groups in **3** is compared with those of silole anion/dianion, germole anion/dianion, and 3-silolenide 2,5-carbodianions **10** and **11**.

## 1. Introduction

Cyclopentadienyl anion, the most representative aromatic compound, has for a long time played important roles in organic and organometallic chemistry [1,2,3]. Therefore it has been a challenge to synthesize the analogue framework [4,5,6,7], in which one of carbon atoms is replaced by a heavier group 14 atom, and the ultimate question is to find out how its aromaticity changes and is maintained [8,9,10,11,12,13,14,15]. Since the first silacyclopentadienide dianion was reported [16], the aromaticity of sila- and germa-cyclopentadienide dianion has been suggested by NMR chemical shift changes upon reduction [17,18]. Their aromatic structures [19,20] and the related structures have been confirmed by X-ray crystallography [21,22,23,24,25,26,27]. Heavier metallic dianion equivalents, the stannacyclopentadienide dianion [28,29,30] and plumbacyclopentadienide dianion [31], are also reported to display aromaticity [32,33,34,35,36,37].

The principal heavier congener of the cyclopentadienide anion, 1-*tert*-butyl-2,3,4,5-tetraphenyl-1-silacyclopentadienide anion, has been reported to have aromaticity according to NMR chemical shift changes upon reduction [38]. Meanwhile 1-methyl-2,3,4,5-tetraphenyl-1-silacyclopentadienide anion was synthesized and crystallized in THF as a [2+2] dimer of its Si = C bond in aromatic ring structures [39], the dimer of which is dissociated to the original silole anions when it is reacted with alkyl halides or trimethylchlorosilane in THF [40]. Even the analogue frameworks of trimetallic anion {[C_2_GeSi_2_]^−^, [C_2_Si_3_]^−^} and divalent germanium containing anion {[C_3_NGe:]^−^}, in which more than one carbon atom of the cyclopentadienyl anion are replaced by heavier group 14 atoms of Si and/or Ge, are synthesized and characterized to have aromaticity [41,42,43], making it possible to form heavy analogues of ferrocene with them [44,45].

In contrast spectroscopic and X-ray crystallographic data [22,23] for 1-trimethylsilyl-tetramethyl/ethyl-1-silacyclopentadienide anions have revealed that they possess pyramidal silicon centers and bond localization in their butadiene moieties. Nevertheless the heavy analogues of ferrocene are synthesized with them [24,27,46,47]. Therefore it is interesting to study 1-trimethylsilyl-2,3,4,5-tetraphenyl-1-silacyclopentadienide anion [Ph_4_C_4_Si(SiMe_3_)]^−^ to compare it with other metallole anions [48].

There are several routes for silole syntheses, via 1,4-dilithio-butadienides by using diphenylacetylene [16,17,49] and 1,4-dihalobutadienes [23,50,51], the intramolecular reductive cyclization of diethynylsilanes [52,53], metallacyclic transfer reactions [54], and organoboration [55,56,57,58]. However those synthetic methods are not applicable to synthesizing of various siloles derivatives at the Si atom, especially for preparing 1-trimethylsilyl group substituted siloles due to the feasibility of the nucleophilic attack on the Si-Si bond by carbanions [22,24,27,59,60] and silole anion [39]. Coversely all metallole dianions are potential and useful intermediates for the synthesis of various di-substituted metallole derivatives, polysiloles, and silole-containing polymers [61,62,63,64,65,66].

Herein we report that silole dianion is a versatile intermediate to synthesize [Ph_4_C_4_Si(SiMe_3_)(R)] (R = Me, Et, *i*-Bu) via [Ph_4_C_4_Si(SiMe_3_)]^−^, which is prepared by hemisilylation of the silole dianion and characterized by ^1^H-, ^13^C-, and ^29^Si-NMR spectroscopy, and [Ph_4_C_4_SiR_2_] (R = *i*-Bu, *t*-Bu).

## 2. Results and Discussion

### 2.1. Preparation of 1-Trimethylsilyl,1-lithio-2,3,4,5-tetraphenyl-1-silacyclopentadienide Anion (**3**) and Its Reaction with Methyl Iodide, Ethyl Iodide and i-Butyl Bromide

**Scheme 1:**
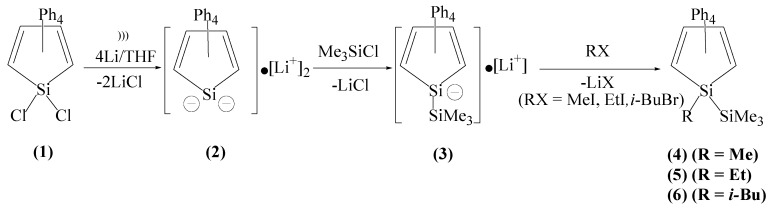
Synthesis and alkylation of **3** via silole dianion **2**.

1-Trimethylsilyl-2,3,4,5-tetraphenyl-1-silacyclopentadienide anion [Ph_4_C_4_Si(SiMe_3_)]^−^•[Li]^+^ (**3**) was prepared from the reaction of the silole dianion [Ph_4_C_4_Si]^−2^•2[Li]^+^ (**2**) with one equivalent of trimethylsilyl chloride. The silole dianion **2** was generated by the sonication of 1,1-dichloro-2,3,4,5-tetraphenyl-1-silacyclopentadiene [Ph_4_C_4_SiCl_2_] (**1**) with lithium in THF [17]. Compound **3** in THF was reacted with the alkyl halides of methyl iodide, ethyl iodide, and *i*-butyl bromide to provide [Ph_4_C_4_Si(SiMe_3_)(R)] [R = Me (**4**) , Et (**5**), *i*-Bu (**6**)], respectively (Scheme 1).

Silylation of **2** with trimethylsilyl chloride is a novel reaction to synthesize 1-trimethylsilyl substituted silole anion **3**; the similar alkylation of stannole dianion with *t*-butyl chloride was reported to give 1-*t*-butyl substituted stannole anion, oxidation of which in the air provided 1,1-bis(1-*t*-butyl-stannole) [67,68]. Oxidation of stannole dianion were reported to give bistannole-1,2-dianion or terstannole-1,3-dianion [69,70]. But the silole anion **3 ** decomposes in the air to give the ring opening products of 1,2,3,4-tetraphenylbutadiene and silicate. Preparation of **4** is interesting since addition of trimethylsilyl chloride to the silole anion [Ph_4_C_4_Si(Me)]^−^•[M]^+^ (M = Li, Na) in THF has given 1,1-bi(1-methyl-silole) [Ph_4_C_4_Si(Me)]_2_, but addition of the silole dianion to trimethylsilyl chloride in THF has provided [Ph_4_C_4_Si(SiMe_3_)(Me)] (**4**) [40].

### 2.2. Synthesis of 1,1-Bis(n-butyl/t-butyl)-2,3,4,5-tetraphenyl-1-silacyclopentadiene and NMR-Study of 3-Silolenide-2,5-carbodianions

**Scheme 2:**
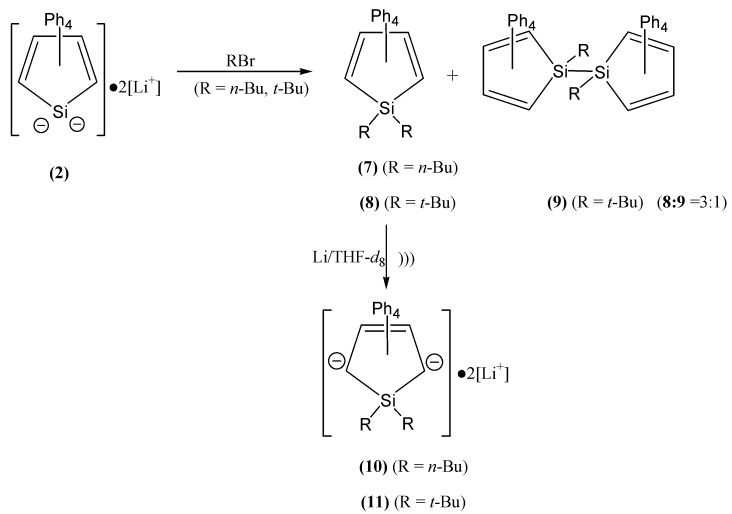
Synthesis of **7** and **8** and their reduction to **10** and **11**.

1,1-bis(*n*-Butyl/*t*-butyl)-2,3,4,5-tetraphenyl-1-silacyclopentadiene {[Ph_4_C_4_Si(*n*-Bu)_2_] (**7**) and [Ph_4_C_4_Si(*t*-Bu)_2_] (**8**)} are prepared in good yield from the reactions of silole dianion **2**, which is generated by the sonication of **1** with lithium in THF, with *n*-bromobutane and *t*-butyl bromide. In the case of *t*-butyl bromide, [Ph_4_C_4_Si(*t*-Bu)_2_] (**8**) is produced along with 1,1-bi[(*t*-Bu)SiC_4_Ph_4_] (**9**) in the ratio of **3** to **1** (Scheme 2). Until now there is one report of the synthesis of 1,1-bis(*t*-butyl)-substituted silole, which has been prepared photochemically in low yield [71].

1,1-bis(*n*-Butyl/*t*-butyl)-2,3,4,5-tetraphenyl-1-silacyclopentadiene {[Ph_4_C_4_Si(*n*-Bu)_2_] (**7**) and [Ph_4_C_4_Si(*t*-Bu)_2_] (**8**)} are sonicated in THF-*d*_8_ with lithium in the 5 mm NMR tube for 2 h. During this time the color of the mixture becomes red and/or purple. The NMR study of the reduced species in THF-*d*_8_ shows clearly that the only one species is formed and is assigned to the respective reduced 3-silolenes with 2,5-carbodianions {[Ph_4_C_4_Si(*n*-Bu)_2_]^−2^•2[Li]^+^ (**10**) and [Ph_4_C_4_Si(*t*-Bu)_2_]^−2^•2[Li]^+^ (**11**)}. Each of their ^13^C-NMR spectra presents ten peaks, consistent with *C*_2_ symmetry, and the ^29^Si spectrum of each compound shows only one resonance. The respective ^1^H-NMR spectrum of **10** and **11** shows two kinds of protons, 20 phenyl protons and 18 butyl protons. Even if they are sonicated further, they show the same peaks with no change.

Both chemical shifts of the two *tert*-C^−^ groups (73.18 ppm for (**10**), 78.12 ppm for (**11**)) are consistent with those of the reported 3-silolenides with 2,5-carbodianions, [Ph_4_C_4_Si(R_1_)(R_2_)]^−2^ (77.4 ppm for R_1_ = R_2_ = Me [72], 76.42 ppm for R_1_ = Me, R_2_ = H [73]), and 1,1-R_1_,R_2_-2-lithio-2,3,4,5-tetraphenyl-1-silacyclopenta-3-enide anion (77.78 ppm for R_1_ = R_2_ = H [74]). The ^13^C-NMR chemical shifts of two C_i__α_, C_i__β_ and two C_p__α_, C_p__β_ show at 151.51, 147.68 ppm and 108.49, 120.80 ppm for **10** and at 152.59, 147.36 ppm and 110.87, 120.25 ppm for **11** (Table 1). The localized carbanions polarize the phenyl groups more than those of the aromatic silole/germole dianions and silole anions. The extent of polarization [Sum(C_i_-C_p_)/2] in those species shows in narrow range: 3-silolenides 2,5-carbodianions (34.42 to 35.00 ppm), silole/germole dianions [Ph_4_C_4_E]^−2^ [E = Si (2), Ge] (28.60 to 28.64 ppm), and silole anion [Ph_4_C_4_Si(*t*-Bu)]^−^ (24.65 ppm). In case of the phenyl group on germanium atom in the localized germole anion [Me_4_C_4_GePh]^−^•[Li]^+^ [75], the extent of polarization {[Sum(C_i_-C_p_)/2] = 35.3 ppm} is very close to those of the localized 3-silolenide 2,5-carbodianions (Table 1 and Table 2).

Upon lithiation of **8** to **11** the ^29^Si-NMR chemical shift of **11** is not changed much (16.49 ppm (**8**) to 13.69 ppm (**11**) since there is no change of its hybridization with the same substituents on the silicon atom (Table 1).

### 2.3. NMR Study of 1-Trimethylsilyl,1-lithio-2,3,4,5-tetraphenyl-1-silacyclopentadienide Anion (**3**) 

[Ph_4_C_4_SiCl_2_] (**1**) is sonicated in THF-*d*_8_ with lithium in a 5 mm NMR tube for 2 h, whereby the color of the mixture becomes red and/or purple. NMR study of the species in THF-*d*_8_ clearly indicates that only one species of silole dianion [Ph_4_C_4_Si]^−2^•2[Li]^+^ (**2**) is generated. Upon adding one equivalent of trimethylsilyl chloride to **2** the ^29^Si-NMR chemical shift changes from 68.54 ppm (for **2**) to −13.22 ppm with another new resonance peak of the trimethylsilyl group at −15.54 ppm {[Ph_4_C_4_Si(SiMe_3_)]^−^•[Li]^+^ (**3**)}. The ^13^C-NMR spectrum of **3** shows ten peaks in the aromatic region, consistent with *C*_2_ symmetry, and one peak for the trimethylsilyl group (Table 1). In its ^1^H-NMR spectrum of it there are two kinds of protons, 20 protons corresponding to four phenyl groups and 9 protons of one trimethylsilyl group.

**Table 1:** ^13^C/^29^Si-NMR chemical shifts of the localized 3-silolenides and germole anions.

3-Silenes 2,5-carbanion	[Ph_4_C_4_SiMe_2_]^−2^•2[Li]^+^	[Ph_4_C_4_SiMeH]^−2^•2[Li]^+^	[Ph_4_C_4_Si( *n*-Bu)_2_]^−2^•2[Li]^+^ (10)	[Ph_4_C_4_Si( *t*-Bu)_2_]^−2^ •2[Li]^+^ (11)	[Me_4_C_4_GePh]^−^•[Li]^+^
C_α_	77.4	76.42	73.18	78.12	138.7
C_β_	128.5	128.82	128.06	130.34	151.5
Sum (C_α_ + C_β_)	205.9	205.24	201.24	208.46	290.2
Sum (C_β_ − C_α_)	51.1	52.40	73.18	78.12	12.8
	Ph	Ph	Ph	Ph	Ph
C_i_	150.6, 147.3	150.33, 147.82	151.51, 147.68	152.59, 147.36	159.6
C_o_	123.3, 125.8	132.47, 126.62	132.88, 126.61	132.75, 127.75	136.4
C_m_	126.5, 132.4	123.05, 125.98	123.58, 126.61	125.77, 125.93	127.3
C_p_	107.8, 120.5	107.65, 120.53	108.49, 120.80	110.87, 120.25	124.3
Sum (C_i_ − C_p_)/2	69.6/2 = 34.8	69.97/2=35.00	69.90/2 = 34.95	68.83/2 = 34.42	35.3^a^
^29^Si-Ring	−	-34.14	-0.27	13.69	−
CH_3_, *tert*-C	−	2.58	14.70, 18.88, 27.34, 29.08	31.5, 33.3 (brd d)	−
Reference	72 ^b^	73 ^b^	This Work ^b^	This Work ^b^	75 ^b^

^b^ In THF-*d*_8_, reference = 25.30 ppm.

**Table 2:** ^13^C/^29^Si-NMR chemical shifts of silole/germole dianions and silole anions.

	[Ph_4_C_4_Si]^−^^2^•2[Li]^+^ (2)	[Ph_4_C_4_Ge]^−^^2^•2[Li]^+^	[Ph_4_C_4_Si( *t*-Bu)]^−^•[Li]^+^	[Ph_4_C_4_SiSiMe_3_]^−^•[Li]^+^ (3)
Ring carbons	151.22, 129.71 ^a^	165.57, 129.92 ^a^	155.76, 139.51	159.67, 139.30
	Ph	Ph	Ph	Ph
C_i_	151.67, 145.83	152.17, 146.30	149.29, 144.72	148.81, 145.72
C_o_	129.97, 133.43	129.92, 133.49	130.50, 132.56	129.81, 132.85
C_m_	126.38, 126.38	126.38, 126.38	126.40, 126.51	126.30, 126.46
C_p_	119.48, 121.83	119.29, 121.91	121.38, 123.34	120.86, 122.86
Sum(C_i_ − C_p_)/2	56.19/2 = 28.10	57.27/2 = 28.64	49.29/2 = 24.65	40.81/2 = 20.41
CH_3_, *tert*-C	−	−	32.78(CH_3_), 23.58(*tert*-C)	-0.23 [Si(CH_3_)_3_]
^29^Si-Ring	68.54	−	25.10	−13.22
Refenence	17 ^b^	18 ^b^	38 ^b^	This Work ^b^

^a^ The reported assignments were revised [76], the chemical shifts did not coincided with each other [77]. ^b^ In THF-*d*_8_, reference = 25.30 ppm.

Upon adding trimethylsilyl chloride to **2** the chemical shifts of C_α_ and C_β_ in **2** are shifted far downfield from 151.22 ppm and 129.71 ppm to 159.67 ppm and 139.30 ppm in **3**. The chemical shifts of C*_i_*_α_ and C*_i_*_β_ in **3** are observed at 145.72 ppm and 148.81 ppm, while the chemical shifts of C_pα_ and C_pβ_ in **3** are observed at 122.86 ppm and 120.86 ppm respectively. These carbon peaks of four phenyl groups indicate that the phenyl groups of **3** are still polarized, and the average chemical shift difference of C_i_ and C_p_ is 20.41 ppm [Sum(C_i_ − C_p_)/2] (Table 2). Such polarizations of phenyl groups are generally observed due to the absence of the significant π-conjugation between their phenyl groups and 5-membered ring because of their bulkiness and the congestion of four phenyl groups. The average chemical shift difference of 20.41 ppm for **3** is smaller than those of the silole dianion [Ph_4_C_4_Si]^−2^•2[Li]^+^ (**2**) (28.10 ppm) [17], [Ph_4_C_4_Si]^−2^•2[Na]^+^ (29.17 ppm) [16], the germole dianion [Ph_4_C_4_Ge]^−2^•2[Li]^+^ (28.64 ppm) [18], and even the silole anion [Ph_4_C_4_Si(*t*-Bu)]^−^•[Li]^+^ (24.65 ppm) [38]. The difference is also significantly smaller than those of the localized 3-silolenes (the reduced siloles to 2,5-carbodianions); [Ph_4_C_4_SiMe_2_]^−2^•2[Li]^+^ (34.8 ppm) [72], [Ph_4_C_4_SiHMe]^−2^•2[Li]^+^ (35.00 ppm) [75], [Ph_4_C_4_Si(*n*-Bu)_2_]^−2^•2[Li]^+^ (34.95 ppm) (**10**), [Ph_4_C_4_Si(*t*-Bu)_2_]^−2^•2[Li]^+^ (34.42 ppm) (**11**), and that of the phenyl group in the localized germole anion [Me_4_C_4_GePh]^−^•[Li]^+^ (35.3 ppm) [75] (Table 1). This trend implies that the electron density in the silole ring carbons of **3** is significantly lower than those in the rings of the localized 3-silolenes, the high aromatic silole/germole dianions {[Ph_4_C_4_Si]^−2^, [Ph_4_C_4_Ge]^−2^} and the silole anion [Ph_4_C_4_Si(*t*-Bu)]^−^ due to its low aromaticity and/or sp^3^ hydridization character on Si atom in **3**.

X-ray crystallographic data for 1-trimethylsilyl-2,3,4,5-tetramethyl/ethyl-1-silacyclopentadienide anion) [R_4_C_4_Si(SiMe_3_)]^−^ (R = Me, Et) have revealed that the anionic rings possess a pyramidal silicon center and bond localization in the butadiene moiety of the ring, the ^29^Si-NMR chemical shifts of these pyramidal ring Si atoms in those anions are observed from −41 ppm to −54 ppm [23]. However in the case of **3** the ^29^Si-NMR chemical shift is observed at −13.22 ppm, far downfield from those of the pyramidal Si atoms in the localized silole anions and far upfield from those of silole dianions and silole anion (Table 3).

**Table 3:** ^29^Si-NMR chemical shifts of silole anions and dianion.

Silole Anion	[Me_4_C_4_SiSiMe_3_]^−^•[M]^+^				[Et_4_C_4_SiSiMe_3_]^−^• [M]^+^		[Et_4_C_4_Si]^−^^2^•2[M]^+^	[Ph_4_C_4_SiSiMe_3_]^−^•[M]^+^ (3)
M	Li		K		Li	K	Li	Li
^29^Si-Ring	−45.38	−43.96	−42.70	−41.52	−53.12 ^c^	−47.38	24.96	−13.22
^29^Si-Ring with crown ether	−	12-CE-4	−	18-CE-6	12-CE-4	−	−	−
^29^Si-SiMe_3_	−12.47	−11.68	−12.44	−11.00	−14.27	−14.22	−	−15.54
Reference	22 ^a^	22 ^b^	22 ^a^	22 ^b^	22 ^c^	22 ^c^	51 ^c^	This work ^c^

^a^ In CH_2_Cl_2_-*d*_2_. ^b^ In Benzene-*d*_6_. ^c^ In THF-*d*_8_.

The ^13^C-NMR and ^29^Si-NMR chemical shifts of **3** do not support its aromaticity, the introduction of trimethylsilyl group on the silicon atom might decrease aromaticity of silole anion with the substituent effect of the trimethylsilyl group enhancing the s-character of the lone pair on the silicon atom and decreasing the s-character of the Si-Si bond in **3** [23,48].

## 3. Experimental

### General Procedures

All reactions were performed under an inert nitrogen atmosphere using standard Schlenk techniques. Air sensitive reagents were transferred in a nitrogen-filled glove box. THF and ether were distilled from sodium benzophenone ketyl under nitrogen. Hexane and pentane were stirred over concentrated H_2_SO_4_ and distilled from CaH_2_. NMR spectra were recorded on JEOL GSX270 and GSX400 spectrometers. GC-MS and solid sample MS data were obtained on a Hewlett-Packard 5988A GC-MS system equipped with a methyl silicon capillary column. Elemental analyses were done by Desert Analytics (Tucson, AZ, USA).

*[Ph_4_C_4_Si(SiMe_3_)(R)]* (R = Me (**4**), Et (**5**), *i*-Bu (**6**)). [Ph_4_C_4_SiCl_2_] (**1**) (0.57 g, 1.25 mmol) was sonicated in THF with an excess of lithium for 5 h. Then the remaining lithium was removed by filtration to give a red-purple solution. The solution was added to methyl iodide in THF with stirring at room temperature for 4 h to give a yellow solution. After removing the solvent under vacuum the remaining yellow solid was extracted with hexane. The concentrated solution was kept in a refrigerator for a couple of days to provide yellow crystals.

*[Ph_4_C_4_Si(SiMe_3_)(Me)]* (**4**). Yield: 0.38 g (65%); mp. 130–132 °C (lit. [40], mp. 130–132 °C).

*[Ph_4_C_4_Si(SiMe_3_)(Et)]* (**5**). Yield: 0.54 g (59%); mp. 100–102 °C, ^1^H-NMR (CDCl_3_, ref; ext. TMS = 0.00 ppm), 0.05 (s, SiMe_3_, 9H), 1.0–1.2 (brd m, ethyl, 5H), 6.68–7.15 (m, 20H); ^13^C-NMR (CDCl_3_, ref; solvent = 77.00 ppm), −1.40 (SiMe_3_), 3.06 (CH_2_), 8.64 (CH_3_); ^29^Si-NMR (CDCl_3,_ ref; ext. TMS = 0.00), −2.53 (ring Si), −16.15 (SiMe_3_); Anal. Calcd. for C_33_H_3__4_Si_2_: C, 81.42; H, 7.04, Found: C, 81.59; H, 7.19.

*[Ph_4_C_4_Si(SiMe_3_)(i-Bu)]* (**6**)*.* Yield: 0.44 g (68%); mp. 154–156 °C, ^1^H-NMR (CDCl_3_, ref; ext. TMS = 0.00 ppm), 0.03 (s, SiMe_3_, 9H), 0.92 (d, CMe_2_, 6H), 1.16 (d, CH_2_, 2H), 1.85 (m, CH, 1H), 6.68–7.15 (m, 20H); ^29^Si-NMR (CDCl_3,_ ref; ext. TMS = 0.00), −6.49 (ring Si), −15.88 (SiMe_3_); Anal. Calcd. for C_35_H_3__8_Si_2_: C, 81.65; H, 7.44, Found: C, 81.76; H, 7.31.

*[Ph_4_C_4_Si(n-Bu)_2_]* (**7**). [Ph_4_C_4_SiCl_2_] (**1**) (0.57 g, 1.25 mmol) was sonicated with an excess of lithium for 5 h. Then the remaining lithium was removed by filtration to give a red-purple solution of the silole dianion. The solution was added to a THF solution of 1-bromobutane with stirring at room temperature for 10 h to give a yellow solution. After removing the solvent under vacuum the remaining yellow solid was extracted with hexane. The concentrated solution was kept in a refrigerator for a couple of days to provide yellow crystals. Yield: 0.56 g (90%); mp. 85 °C (lit. [78] mp. 81 °C).

*[Ph_4_C_4_Si(t-Bu)_2_]* (**8**). [Ph_4_C_4_SiCl_2_] (**1**) (0.55 g, 1.21 mmol) was sonicated with an excess of lithium in THF for 5 h. Then the remaining lithium was removed by filtration to give a red-purple solution of **2**. The solution was added to a THF solution of *t*-butyl bromide with stirring at room temperature for 24 h to give a yellow solution. After removing the solvent under vacuum the remaining yellow solid was extracted with ether. The concentrated solution was kept in a refrigerator for a couple of days to provide pale yellow crystals of bissilole 1,1-bi[(*t*-Bu)SiC_4_Ph_4_] [14]. The filtered solution was concentrated under vacuum, then it was kept in a refrigerator for a couple of days to give yellow crystals of [Ph_4_C_4_Si(*t*-Bu)_2_] (**8**). Yield: 0.33 g (54%); mp. 169-171 °C, ^1^H-NMR (CDCl_3_, ref; ext. TMS = 0.00 ppm), 1.16 (s, Me, 18H), 6.68–7.15 (m, 20H), ^29^Si-NMR (CDCI_3,_ ref; ext. TMS=0.00), 16.49 (ring Si); Anal. Calcd. for C_36_H_38_Si_1_: C,86.69; H,7,68, Found: C, 86.71; H, 7,75.

*1,1-Bi[(t-Bu)SiC_4_Ph_4_]* (**9**)*.* Yield: 0.19 g (18%); mp. 295–307 °C (lit. [16] mp. 296-307 °C), ^29^Si-NMR (THF-*d*8_,_ ref; ext. TMS = 0.00), 3.62 (ring Si).

*1,1-Bis(R)-2,5-dilithio-2,3,4,5-tetraphenyl-1-silacyclopenta-3-enide* *anion* [R = *n*-Bu (**10**), R = *t*-Bu (**11**)]. The respective [Ph_4_C_4_Si(*n*-Bu)_2_] (**7**) (0.025 g, 0.05 mmol) and [Ph_4_C_4_Si(*t*-Bu)_2_] (**8**) (0.025 g, 0.05 mmol) was transferred into 5 mm NMR tube, they were sonicated with an excess of lithium in THF-*d*_8_ for 2 h to give a red-purple solution. Then ^1^H-, ^13^C-, and ^29^Si-NMR spectroscopic study was performed.

*[Ph_4_C_4_Si(n-Bu)_2_]^-2^•2[Li]^+^* (**10**); ^1^H-NMR (THF-*d*_8_, ref; ext. TMS = 0.00 ppm), 0.83 (t, CH_3_, 6H), 0.90 (m, CH_2_, 4H), 1.36 (sept, CH_2_, 4H), 1.52 (m, CH_2_, 4H), 6.68–7.15 (m, 20H), ^29^Si-NMR (THF-*d*_8__,_ ref; ext. TMS = 0.00), −0.27 (ring Si). *[Ph_4_C_4_Si(t-Bu)_2_]^-2^•2[Li]^+^* (**11**); ^1^H-NMR (THF-*d*_8_, ref; ext. TMS = 0.00 ppm), 1.21 (brd s, Me, 18H), 6.68-7.15 (m, 20H), ^29^Si-NMR (THF-*d*_8__,_ ref; ext. TMS = 0.00), 13.69 (ring Si).

## 4. Conclusions

Silole dianion [Ph_4_C_4_Si]^−2^ (**2**) is a versatile intermediate to prepare symmetrically substituted siloles of [Ph_4_C_4_SiR_2_] (R = *n*-Bu, *t*-Bu) and unsymmetrically substituted siloles of [Ph_4_C_4_Si(SiMe_3_)(R)] (R = Me, Et, *i*-Bu). The formers are synthesized from the reaction of silole dianion **2** with the corresponding alkyl bromides, while the latter are synthesized via [Ph_4_C_4_Si(SiMe_3_)]^−^•[Li] ^+^ (**3**) by hemsilylation of **2** with trimethylsilyl chloride and then by alkylation of **3** with the corresponding alkyl halides. The silole anion **3** and the reduced 3-silolenide 2,5-carbodianions {[Ph_4_C_4_Si(*n*-Bu)_2_]^−^^2^•2[Li]^+^ (**10**) and [Ph_4_C_4_Si(*t*-Bu)_2_]^−^^2^•2[Li]^+^ (**11**)} are characterized by ^1^H-, ^13^C-, and ^29^Si-NMR spectroscopy.
